# Thiol-reducing agents prevent sulforaphane-induced growth inhibition in ovarian cancer cells

**DOI:** 10.1080/16546628.2017.1368321

**Published:** 2017-08-28

**Authors:** Seung Cheol Kim, Boyun Choi, Youngjoo Kwon

**Affiliations:** ^a^ Department of Medical Science, Ewha Womans University, Seoul, Republic of Korea; ^b^ Department of Food Science and Engineering, Ewha Womans University, Seoul, Republic of Korea

**Keywords:** Sulforaphane, reactive oxygen scavengers, thiol-reducing agents, diamide, ovarian cancer

## Abstract

The inhibitory potential of sulforaphane against cancer has been suggested for different types of cancer, including ovarian cancer. We examined whether this effect is mediated by mitogen-activated protein kinase (MAPK) and reactive oxygen species (ROS), important signaling molecules related to cell survival and proliferation, in ovarian cancer cells. Sulforaphane at a concentration of 10 μM effectively inhibited the growth of cancer cells. Use of specific inhibitors revealed that activation of MAPK pathways by sulforaphane is unlikely to mediate sulforaphane-induced growth inhibition. Sulforaphane did not generate significant levels of intracellular ROS. Pretreatment with thiol reducers, but not ROS scavengers, prevented sulforaphane-induced growth inhibition. Furthermore, diamide, a thiol-oxidizing agent, enhanced both growth inhibition and cell death induced by sulforaphane, suggesting that the effect of sulforaphane on cell growth may be related to oxidation of protein thiols or change in cellular redox status. Our data indicate that supplementation with thiol-reducing agents should be avoided when sulforaphane is used to treat cancer.

## Introduction

Sulforaphane is a dietary isothiocyanate that is present as a glucosinolate precursor in cruciferous vegetables, including broccoli and cauliflower. Sulforaphane is produced from glucoraphanin, a glucosinolate, by the action of myrosinase, which is released upon damage to plants [1] or by intestinal microflora [2]. Young broccoli and cauliflower sprouts are particularly high in glucoraphanin [3].

Epidemiological studies have suggested that a high level of consumption of cruciferous vegetables reduces the risk of many types of cancer [4–6], and the protective effect of crucifers against cancer has been attributed to their high glucosinolate content [1,4]. Sulforaphane is a glucosinolate derivative that has been widely studied for its anticancer activity [4,7]. Sulforaphane has been demonstrated to induce phase II detoxification and antioxidant enzymes [8] and to inhibit phase I enzymes that activate pro-carcinogens [9]. Administration of sulforaphane inhibits and/or retards tumorigenesis induced by carcinogens in animal models [10,11]. In addition to its preventive effect on cancer, sulforaphane has recently been shown to inhibit the growth of various types of cancer cells by modulating multiple pathways related to cancer progression [7,12].

Ovarian cancer is one of the major types of cancer that affect female reproductive organs. The GLOBOCAN 2012 estimated that 0.32 million new ovarian cancer cases and 0.15 million cancer-related deaths occurred worldwide in 2012 [13]. Ovarian cancer is more prevalent in developed countries than in developing countries and has the highest mortality rate among gynecological malignancies [13]. Rapid progression without symptoms complicates the clinical management of ovarian cancer [14]. Moreover, most ovarian cancer patients experience disease relapse due to drug resistance [15,16]. One promising cancer management strategy can be the use of bioactive compounds derived from food, alone or in combination with existing chemotherapeutic treatments, to increase therapeutic efficacy [17]. Thus, it is important to identify the molecular mechanisms of bioactive compounds in food in order to identify cancer patients who may benefit from food-derived compounds and to establish combination strategies with available therapeutic drugs or other bioactive compounds found in food [17].

Sulforaphane has been suggested to have inhibitory effects in ovarian cancer, but sulforaphane-mediated anticancer mechanisms have not been fully described. Sulforaphane effectively reduces activation of the AKT signaling pathway in ovarian cancer cells that constitutively overexpress AKT [12]. Sulforaphane also induces cell-cycle arrest in PA-1 cells [18] and in MDAH 2774 and SKOV3 cells [19].

The cancer-preventive effect of sulforaphane has been primarily attributed to its antioxidative activity [8]. However, its therapeutic effect is suggested to be mediated by generation of reactive oxygen species (ROS) in leukemia [20] and bladder [21] and prostate [22] cancer. Sulforaphane has also been shown to modulate mitogen-activated protein kinase (MAPK) pathways. Treatment with sulforaphane leads to cell-cycle arrest and apoptosis in pancreatic cancer cells through the inhibition of extracellular signal-regulated kinase (ERK) pathways [23]. In Caco-2 cells, cell-cycle arrest induced by sulforaphane treatment is mediated through ERK but not c-Jun NH_2_-terminal kinase (JNK) [24]. However, some studies have indicated that MAPK modulation by sulforaphane is not directly related to cell death or proliferation of cancer cells; instead, the effects of sulforaphane are attributed to the induction of antioxidant-related genes [25–27]. Moreover, signaling pathways induced by sulforaphane may also depend on the dose of agent [28].

MAPKs and ROS are important cellular mediators that regulate cell survival and proliferation [29]. In addition, modulation of MAPK activation and ROS generation are important therapeutic pathways [30]. Therefore, it is important to determine whether MAPK pathways and ROS generation contribute to the sulforaphane-mediated therapeutic effect. In this study, we evaluated the ability of sulforaphane to inhibit the growth of ovarian cancer cells as well as the involvement of MAPK activation and ROS production in sulforaphane-induced growth inhibition to exploit its therapeutic potential.

## Materials and methods

### Cell lines and cell cultures

The cell lines used were OVCAR3, OVCAR4, OVCAR5, and SKOV3 [14,31] (human ovarian cancer cells) and IHFNO-303 and IHFOT-208 [14] (human ovarian fibroblasts); they were kindly gifted by Dr Andrew Godwin (University of Kansas Medical Center, KS, USA). Ovarian cancer cells were maintained in RPMI 1640 medium supplemented with 10% fetal bovine serum (FBS), 0.3 U/ml insulin, 2 mM L-glutamine, 100 U/ml penicillin, and 100 µg/ml streptomycin. Fibroblasts were maintained in Dulbecco’s modified Eagle’s medium supplemented with 10% FBS, 100 U/ml penicillin, and 100 µg/ml streptomycin. Cells were cultured in a humidified incubator at 37°C and 5% carbon dioxide. All reagents were purchased from Thermo Fisher Scientific (Waltham, MA, USA), except for insulin (Life Technologies, Carlsbad, CA, USA).

### Antibodies and inhibitors

Primary antibodies against phospho-p38, p38, phospho-ERK1/2, ERK1/2, and glyceraldehyde-3-phosphate dehydrogenase (GAPDH) were purchased from Cell Signaling Technology (Danvers, MA, USA). Anti-p-JNK and anti-JNK were purchased from Santa Cruz Biotechnology (Dallas, TX, USA). Sulforaphane (DL-sulforaphane) was purchased from Sigma-Aldrich (St. Louis, MO, USA). The inhibitors used were *N*-acetyl cysteine (NAC), dithiothreitol (DTT), 6-hydroxy-2,5,7,8-tetramethylchromane-2-carboxylic acid (Trolox), butylated hydroxyanisole (BHA), and diazenedicarboxylic acid bis(*N*,*N*-dimethylamide) (diamide), all of which were purchased from Sigma-Aldrich. SB203580 and PD98059 were purchased from SelleckChem (Houston, TX, USA). All reagents were dissolved in dimethyl sulfoxide (DMSO) except DTT and BHA, which were freshly prepared in water. The final concentration of DMSO was less than 0.05%.

### Cell viability assay

Cells (2000–3500 cells per well depending on the cell line) were cultured overnight in flat-bottomed 96-well plates. Cell viability was assessed after the addition of sulforaphane at the indicated concentration or vehicle control (0.05% DMSO) for 72 h. At the end of the treatment, 3-[4,5-dimethylthiazol-2-yl]-2,5-diphenyl tetrazolium bromide (MTT) (Biovision, Milpitas, CA, USA) was added and the plates were incubated for a further 4 h. During the 72 h cell viability assay, the medium was not changed. The number of viable cells was estimated by the formation of formazan product as a result of conversion of MTT by viable cells using a VersaMax enzyme-linked immunoassay microplate reader (Molecular Devices, Sunnyvale, CA, USA). To evaluate whether inhibitors of specific pathways prevented sulforaphane-induced cell death, cells were incubated with the inhibitors for 1 h before treatment with sulforaphane.

### Colony formation assay

Cells (800–1400 cells) were cultured overnight in 6-well plates and treated with sulforaphane in concentrations ranging from 0 to 25 μM for 14 days until cells treated with vehicle (0 μM) formed good-sized colonies consisting of more than 200 cells. During the 14 day assay, the medium was not changed and no additional sulforaphane was added. Cells were fixed with 4% paraformaldehyde and stained with 0.2% crystal violet. The total number of colonies was counted using ImageJ software [32].

### Western blot analysis

Cells were lysed using radioimmunoprecipitation assay buffer [50 mM Tris HCl, pH 8.0, 5 mM ethylenediaminetetraacetic acid (EDTA), 150 mM NaCl, 1% NP-40, 0.5% sodium deoxycholate, 0.1% sodium dodecyl sulfate (SDS), 10 mM sodium fluoride, 1 mM sodium orthovanadate, 1 mM β-glycerophosphate] supplemented with protease inhibitor cocktail (Sigma-Aldrich). Cell debris was eliminated by centrifugation at 13,000 rpm for 10 min. The protein concentration in the resulting supernatant was measured using a Bio-Rad protein assay kit (Hercules, CA, USA), and protein samples were stored at −80°C until analysis. The cell lysates were mixed with reducing agents and boiled for 5 min. Equal amounts (35 μg) of denatured proteins were separated by 10% SDS–polyacrylamide gel electrophoresis and subjected to immunoblotting using specific primary antibodies. Protein detection was achieved using LumiFlash Infinity Chemiluminescence Substrate (Visual Protein Biotechnology Co., Taiwan).

### Intracellular ROS measurement

Attached cells (10,000 cells per well in a 96-well plate) were washed with phosphate-buffered saline (PBS) and incubated for 1 h with cell-permeable 2ʹ,7ʹ-dichlorodihydrofluorescin diacetate (DCFH-DA) (Cell Biolabs, San Diego, CA, USA) according to the manufacturer’s instructions. Highly fluorescent DCF, which is produced as a result of oxidation of DCFH by cellular ROS, was measured using a Microplate Fluorometer (Thermo Fisher Scientific).

### Apoptosis analysis

Cells were treated with sulforaphane, diamide, or vehicle for 24 h. After treatment, the cells were harvested and washed with PBS. The cells were suspended in 100 μl of binding buffer and stained with 5 μl annexin V (Thermo Fisher Scientific) and propidium iodide for 15 min. A total of at least 10,000 events were collected and analyzed using BD FACSCalibur (BD Biosciences, San Diego, CA, USA).

### Thioredoxin reductase activity assay

Cells were treated with sulforaphane or vehicle for 24 h. For each treatment, 40 μg of protein was used to measure thioredoxin reductase activity using a thioredoxin reductase activity colorimetric assay kit (Biovision) according to the manufacturer’s instructions.

### Statistical analyses

One-way analysis of variance (ANOVA) was performed using SAS software (version 9.3; SAS Institute, Cary, NC, USA) to determine whether sulforaphane-induced cytotoxicity was altered by treatment with specific inhibitors. If statistically significant differences were found, Tukey’s range test was performed to identify differences. One-way ANOVA was performed to determine whether there was a difference between treatments in cell death rate. A *p* value less than 0.05 was considered significant.

## Results

### Sulforaphane effectively inhibits cell growth in ovarian cancer cells

**Figure 1.**
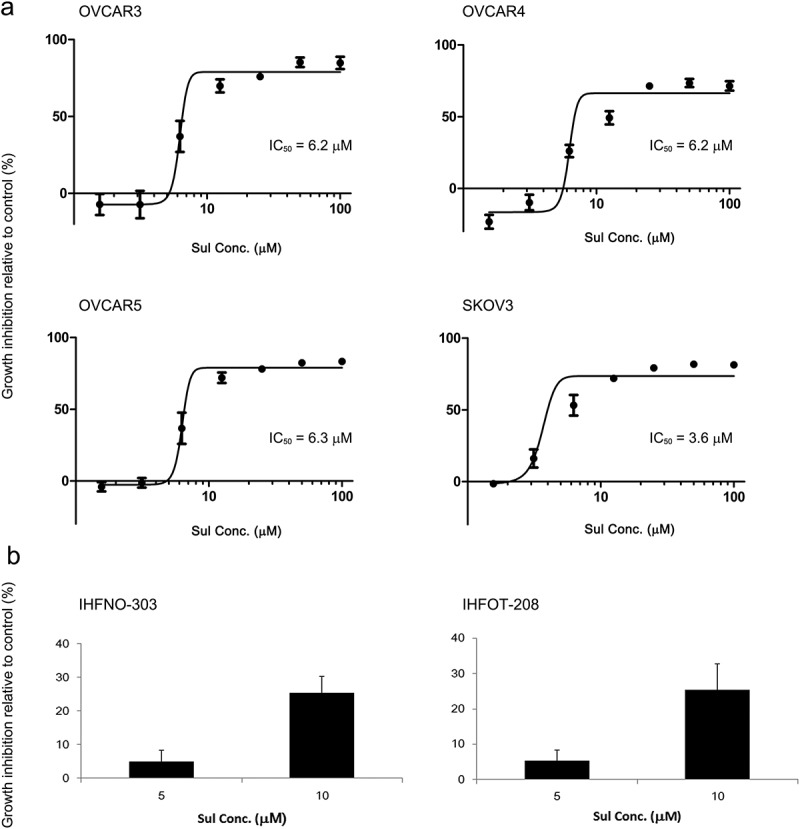
Inhibition of cell viability and/or growth of ovarian cancer cells by treatment with sulforaphane (Sul). (A) OVCAR3, OVCAR4, OVCAR5, and SKOV3 cells were cultured in 96-well plates overnight and then treated with 0–100 μM sulforaphane for 72 h. (B) IHFNO-303 and IHFOT-208 fibroblasts were plated in 96-well plates overnight and then treated with 0, 5, and 10 μM sulforaphane for 72 h. Cell growth was measured using an MTT assay. Cell growth inhibition relative to the vehicle control (% inhibition) is shown. IC_50_ = half maximal inhibitory concentration. Values are mean ± SE, *n* = 3 or 4.

Ovarian cancer cells were treated with sulforaphane at concentrations ranging from 0 to 100 μM, and the effect on cell growth was evaluated. Sulforaphane effectively reduced cell growth at 72 h in all ovarian cancer cells tested (Figure 1(A)). The half maximal inhibitory concentration (IC_50_) was similar with 6.2 or 6.3 μM in OVCAR3, OVCAR4, and OVCAR5, and lower in SKOV3 cells (IC_50_ = 3.6 μM) (Figure 1(A)). Sulforaphane treatment was very effective at 12.5 μM, reaching maximum growth inhibition (Figure 1(A)). Therefore, concentrations of 5–10 μM were considered to be effective in growth inhibition of cancer cells.

We examined how these concentrations of sulforaphane affect the growth of non-cancer cells. Sulforaphane treatment also inhibited the growth of fibroblasts, but did so less effectively; the growth inhibition rate was less than 10% at 5 μM and about 30% at 10 μM (Figure 1(B)). Treatment with 25 μM sulforaphane also induced significant growth inhibition (≥ 50%) in fibroblasts (data not shown). Thus, concentrations of 6.25 and 12.5 μM or 5 and 10 μM were used throughout the study.

Since the MTT assay depends on the activity of mitochondrial reductase to convert tetrazolium dye to insoluble formazan, we also performed a colony formation assay and showed that sulforaphane treatment inhibited cell viability at all concentrations tested, as determined by the total number of colonies formed (supplementary Figure S1).

### Sulforaphane-induced MAPK activation does not mediate sulforaphane-induced growth inhibition of ovarian cancer cells

**Figure 2.**
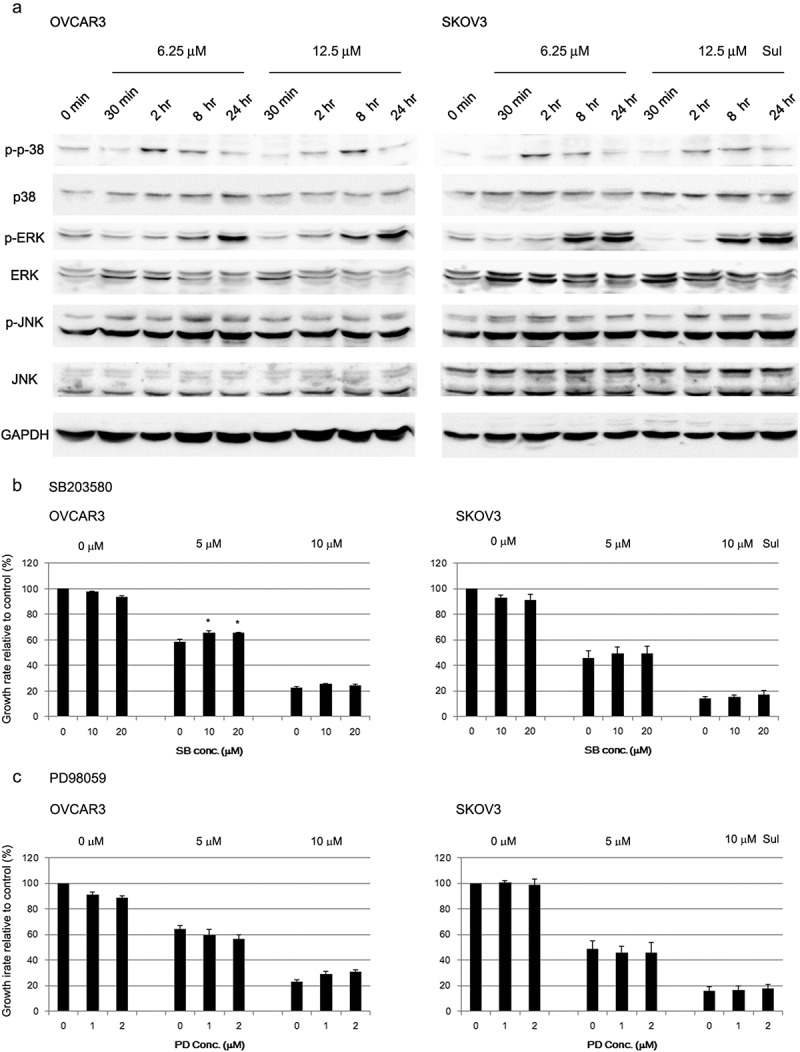
Activation of mitogen-activated protein kinases (MAPKs) by sulforaphane (Sul) in ovarian cancer cells. (A) Activation of MAPKs (p38; ERK, extracellular signal-regulated kinase; JNK, c-Jun NH_2_-terminal kinase; GAPDH, glyceraldehyde-3-phosphate dehydrogenase; p-, phospho-) in OVCAR3 and SKOV3 cells was assessed by immunoblotting after treatment with 6.25 or 12.5 μM sulforaphane. The data shown are representative of three independent experiments. (B) OVCAR3 and SKOV3 cells were pretreated for 1 h with SB203580 (SB), a specific inhibitor of p38 MAPK, before addition of 5 or 10 μM sulforaphane to the media. Growth rate relative to vehicle control is shown. Values are mean ± SE, *n* = 3. An asterisk (*) indicates a significant difference (*p* < 0.05) in the cell growth rate compared to treatment with sulforaphane alone (0 μM SB203580). (C) OVCAR3 and SKOV3 cells were pretreated with a specific inhibitor of extracellular signal-regulated kinase MAPK, PD98059 (PD), before the addition of sulforaphane. Growth rate relative to vehicle control is shown. Values are mean ± SE, *n* = 3.

We examined whether MAPK activation is involved in sulforaphane-mediated growth inhibition. Treatment of ovarian cancer cells with sulforaphane induced activation of both p38 and ERK. Sulforaphane treatment increased phosphorylation of p38 within 2–8 h after treatment, and phosphorylation of ERK was increased after 8 h of sulforaphane treatment in both cell lines (Figure 2(A)). However, activation of ERK and p38 MAPK did not show a dose-dependent increase. Both OVCAR3 and SKOV3 cells constitutively expressed high levels of phosphorylated JNK, and sulforaphane did not significantly alter phospho-JNK expression during the time-course (Figure 2(A)).

Inhibitor concentrations that effectively reduce activation of the corresponding MAPK were used in subsequent studies. Pretreatment with a p38 inhibitor, SB203580, partially prevented the inhibition of cell growth by 5 μM sulforaphane in OVCAR3 cells, but the preventive effect was not significant in SKOV3 cells (Figure 2(B)). Pretreatment with SB203580 did not affect the inhibition of cell growth induced by 10 μM sulforaphane in either OVCAR3 or SKOV3 cells (Figure 2(B)). An MAPK/ERK kinase (MEK) inhibitor, PD98059, did not prevent or sensitize cells to sulforaphane-induced growth inhibition at any concentration tested, although treatment with PD98095 alone slightly (by less than 10%) inhibited cell growth (Figure 2(C)).

### NAC effectively prevents sulforaphane-induced inhibition of cell growth

**Figure 3.**
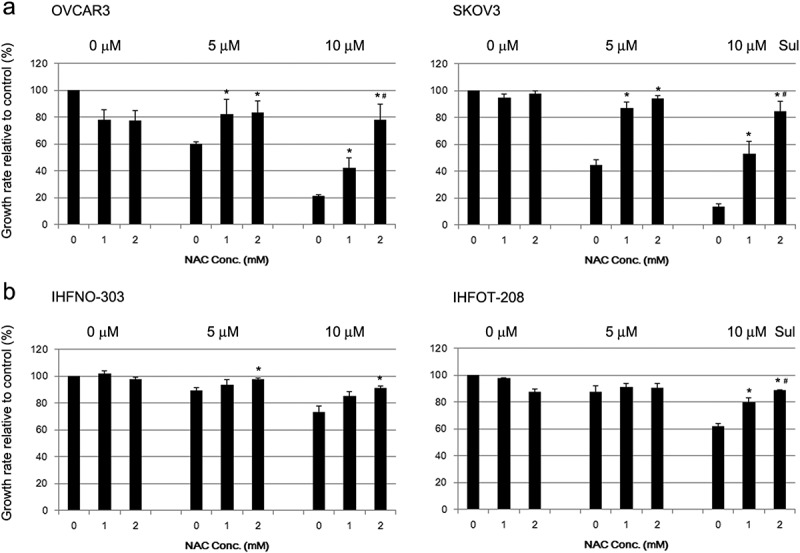
Abrogation of sulforaphane-induced growth inhibition by pretreatment with *N*-acetyl cysteine (NAC). (A) OVCAR3 and SKOV3 cells or (B) IHFNO-303 and IHFOT-208 fibroblasts were preincubated with 1 or 2 mM NAC before treatment with 5 or 10 μM sulforaphane (Sul). Growth rate relative to vehicle control is shown. Values are mean ± SE, *n* = 3. An asterisk (*) indicates a significant difference (*p* < 0.05) in the cell growth rate compared to treatment with sulforaphane alone (0 mM NAC), and a hash sign (#) indicates a significant difference (*p* < 0.05) compared to treatment with a combination of sulforaphane and 1 mM NAC.

We next assessed the involvement of ROS in sulforaphane-induced growth inhibition. NAC, which is widely used as an ROS-scavenging agent, effectively suppressed sulforaphane-induced inhibition of cancer cell growth (Figure 3(A)). The effect of NAC alone was minimal in SKOV3 cells, whereas NAC treatment (2 mM) alone inhibited (by about 20%) cell growth in OVCAR3 cells (Figure 3(A)). However, NAC dose-dependently prevented sulforaphane-induced growth inhibition at 10 μM sulforaphane, which caused approximately 80% growth inhibition, and 2 mM NAC completely abrogated sulforaphane-induced inhibition of cell growth in both OVCAR3 and SKOV3 cells (Figure 3(A)).

Pretreatment with NAC also reduced the sulforaphane-induced decrease in fibroblast growth, but less effectively than in cancer cells (Figure 3(B)). NAC treatment alone slightly decreased the cell proliferation rate (4–10%) in fibroblasts, at a concentration of 2 mM (Figure 3(B)).

### Thiol-reducing agents, but not free radical scavengers, prevent sulforaphane-induced inhibition of cancer cell growth

**Figure 4.**
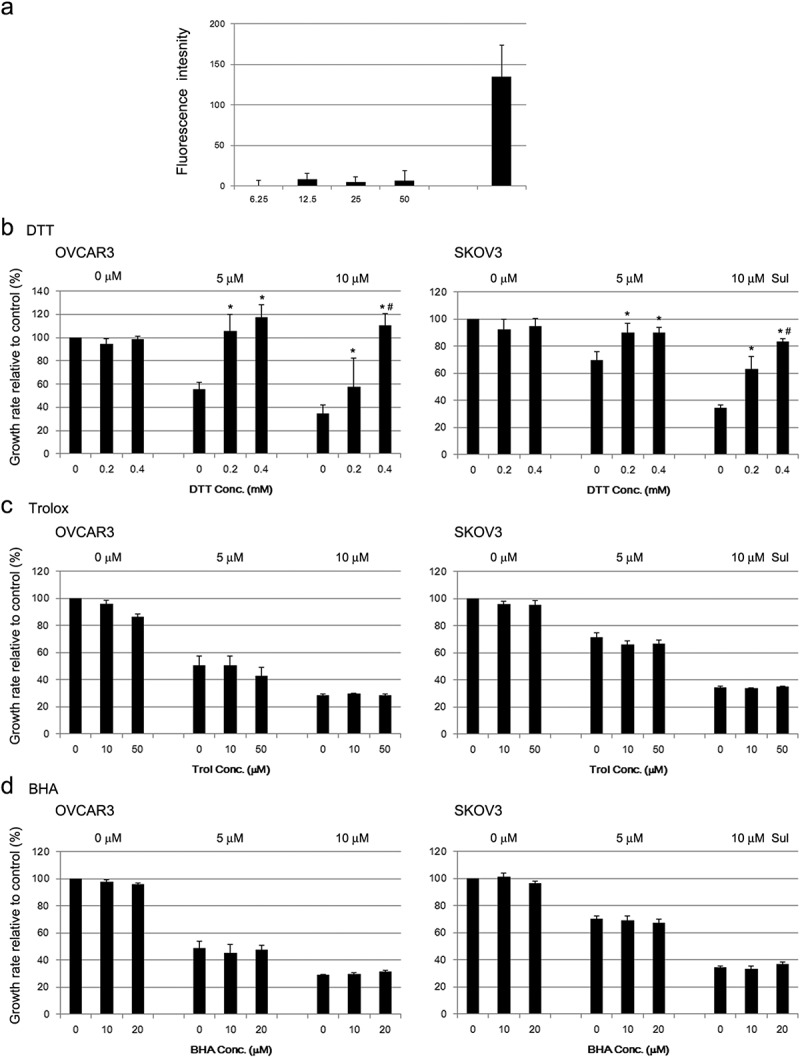
Abrogation of sulforaphane (Sul)-induced inhibition of cell growth by thiol-reducing agents in ovarian cancer cells. (A) SKOV3 cells were plated in a 96-well plate and incubated for 1 h with cell-permeable 2ʹ,7ʹ-dichlorodihydrofluorescin diacetate (DCFH-DA). Fluorescence derived from highly fluorescent DCF as a result of oxidation of DCFH by cellular reactive oxygen species was measured using a microplate reader. Hydrogen peroxide (40 μM) was used as a positive control. Values are mean ± SE, *n* = 3. (B) OVCAR3 and SKOV3 cells were incubated with 0.2 and 0.4 mM dithiothreitol (DTT), respectively, for 1 h and then treated with 5 or 10 μM sulforaphane. Growth rate relative to vehicle control was estimated after 72 h using the MTT assay. Values are mean ± SE, *n* = 3. An asterisk (*) indicates a significant difference (*p* < 0.05) in the cell growth rate compared to treatment with sulforaphane alone (0 mM DTT) and a hash sign (#) indicates a significant difference (*p* < 0.05) compared to treatment with a combination of sulforaphane and 0.2 mM DTT. (C) OVCAR3 and SKOV3 cells were preincubated with 10 or 50 μM Trolox (Trol), and the effect on sulforaphane-induced cell growth inhibition was evaluated, as described in (B). Growth rate relative to vehicle control is shown. Values are mean ± SE, *n* = 3. (D) All conditions were the same as described in (B) except for pretreatment with 10 or 20 μM butylated hydroxyanisole (BHA).

Since NAC was effective in antagonizing sulforaphane-induced growth inhibition of cancer cells, we examined whether sulforaphane induces cellular ROS generation. Sulforaphane did not noticeably induce intracellular ROS at any concentration tested, in contrast to a marked increase in ROS after treatment with 40 μM hydrogen peroxide in SKOV3 cells (Figure 4(A)). Methanol (the vehicle for DCFH-DA, 0.5%) was highly toxic to OVCAR3 cells and prevented the estimation of intracellular ROS levels because of the absence of viable cells after treatment with DCFH-DA.

Because NAC has thiol-reducing capability in addition to its antioxidant effect, we used another thiol-reducing agent, DTT, and non-thiol antioxidants, Trolox and BHA, to examine whether the thiol-reducing property of NAC is critical for abrogating sulforaphane-induced cytotoxicity. Treatment with DTT resulted in an effect that was similar to that of NAC; 0.4 mM DTT almost completely abrogated sulforaphane-induced cytotoxicity in both cell lines (Figure 4(B)), whereas Trolox and BHA did not prevent the growth inhibition induced by sulforaphane (Figure 4(C,D)). Therefore, the thiol-reducing agents NAC and DTT prevented the growth inhibition induced by sulforaphane, but non-thiol antioxidants did not. Treatment with DTT or Trolox alone slightly (by less than 10%) inhibited the cell proliferation rate (Figure 4(B,C)), whereas BHA treatment alone did not affect cell growth (Figure 4(D)).

**Figure 5.**
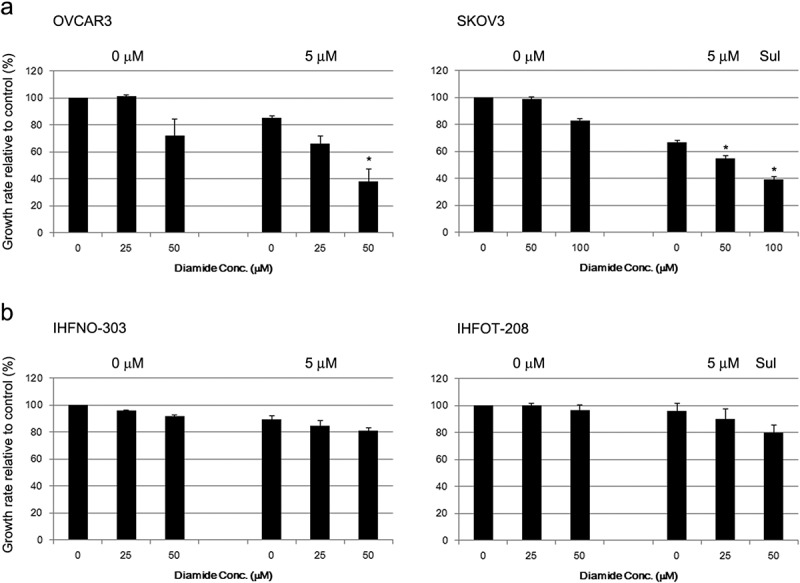
Enhancement of sulforaphane (Sul)-induced inhibition of cell growth by diamide. (A) OVCAR3 and SKOV3 cells were pretreated with diamide (25–100 μM) 1 h before treatment with 5 μM sulforaphane. (B) IHFNO-303 and IHFOT-208 fibroblasts were pretreated with 25 or 50 μM diamide 1 h before treatment with 5 μM sulforaphane. Growth rate relative to vehicle control was estimated after 72 h using the MTT assay. Values are mean ± SE, *n* = 3. An asterisk (*) indicates a significant difference (*p* < 0.05) in the cell growth rate compared to treatment with sulforaphane alone.

**Figure 6.**
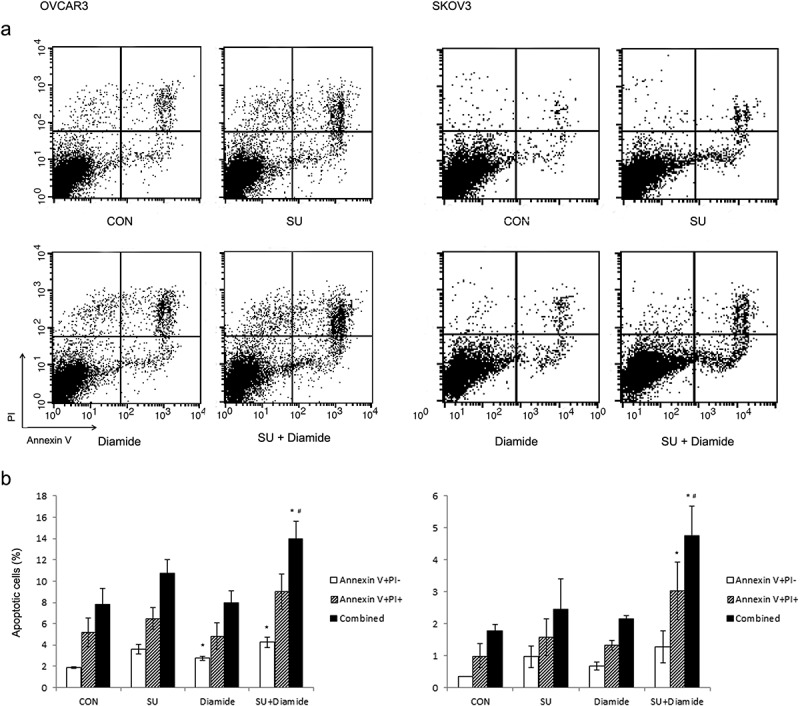
Enhancement of sulforaphane-induced cell death by diamide. OVCAR3 and SKOV3 cells were pretreated with diamide (50 or 100 μM) for 1 h before treatment with 6.25 μM sulforaphane (SU). Cells were stained with Annexin V and propidium iodide (PI). Apoptotic cells were detected using flow cytometry. (A) Representative density plots of dual annexin/PI staining are shown. (B) The percentage of apoptotic cells is shown. Lower right quadrant (annexin V-positive and PI-negative) indicates early apoptotic cells. Upper right quadrant (annexin V and PI-positive) represents necrotic or late apoptotic cells. Both early and late apoptotic cells (Combined) were calculated as the incidence of apoptotic cell death. Values are mean ± SE, *n* = 3. An asterisk (*) indicates a significant difference (*p* < 0.05) in the cell death rate compared to vehicle control and a hash sign (#) indicates a significant difference (*p* < 0.05) compared to treatment with diamide.

We further examined whether sulforaphane-induced growth inhibition is enhanced by treatment with diamide, which oxidizes the thiols of reduced glutathione and of proteins containing free sulfhydryl groups [33]. Treatment with 25 μM diamide alone did not alter cell growth rate, whereas cell proliferation decreased (by 20–30%) with 50 μM diamide (Figure 5(A)). Combination treatment of diamide significantly enhanced the cell growth inhibition induced by 5 μM sulforaphane treatment in OVCAR3 and SKOV3 cells (Figure 5(A)). However, the effect of sulforaphane was not significantly altered by combination treatment of diamide in fibroblasts (Figure 5(B)). In addition, the cell death induced by sulforaphane (6.25 μM) treatment was enhanced by combination treatment with diamide in cancer cells (Figure 6(A)). Combination treatment with sulforaphane and diamide significantly increased the percentage of apoptotic cells compared to vehicle control in OVCAR3 (7.8% vs 14.0% apoptotic cells) and SKOV3 cells (1.8% vs 4.8% apoptotic cells) (Figure 6(B)). Hence, the growth-inhibitory effect of sulforaphane may be related to the oxidation of protein thiols or change in cellular redox status.

We examined whether sulforaphane modulates the activity of thioredoxin reductase, which is responsible for the formation of reduced disulfide bonds in cells [34]. In OVCAR3 cells, 5 μM sulforaphane significantly increased thioredoxin reductase activity, whereas there was no significant change with 10 μM sulforaphane. In SKOV3 cells, no significant change was observed following sulforaphane treatment at any concentration tested (supplementary Figure S2). Therefore, modulation of thioredoxin reductase activity does not appear to be responsible for thiol oxidation.

## Discussion

Modulation of ROS generation and MAPK activation are important therapeutic pathways, and delineation of the effect of sulforaphane on these pathways may be important in the therapeutic use of dietary sulforaphane as a single or combination therapy. The cancer-preventive effect of sulforaphane has been partly attributed to its ability to induce antioxidant enzymes and detoxifying enzymes [35]. It is important to understand how antioxidants modulate the effect of sulforaphane if ROS generation is involved in the sulforaphane-induced inhibition of cancer cell growth, since vegetables that are rich in glucosinolates also contain high concentrations of antioxidants. Therefore, this study investigated whether MAPK signaling pathways and ROS generation are involved in the sulforaphane-induced therapeutic effect on ovarian cancer cells.

Sulforaphane treatment effectively reduced cell viability; IC_50_ values for sulforaphane treated for 72 h were between 3.6 and 6.3 μM, and treatment with 12.5 μM sulforaphane induced maximal growth inhibition in the ovarian cancer cells tested (Figure 1(A)). However, sulforaphane treatment was less effective on non-cancer cells (e.g. fibroblasts). Treatment with 10 μM sulforaphane induced about 30% growth inhibition in IHFNO-303 and IHFOT-208 fibroblasts (Figure 1(B)). Mesenchymal phenotype or other differences compared to cancer cells, such as susceptibility to oxidation [36], may contribute to this result. Chang et al. also reported that 12.5 μM sulforaphane is effective in reducing cell viability of the ovarian cancer cell line PA-1 [18]. In addition, treatment with 8 μM sulforaphane for 72 h effectively reduced growth (by 50%) of the ovarian cancer cell lines MDAH 2774 and SKOV3 [19]. A previous study demonstrated that sulforaphane effectively suppresses the growth of human and mouse ovarian cancer cells that overexpress AKT [12]. In our study, the growth-inhibitory effect of sulforaphane was also observed in OVCAR4 and OVCAR5 cells that express low levels of AKT, in addition to OVCAR3 and SKOV3 cells that express high levels of AKT [14]. Therefore, the effect of sulforaphane was similar regardless of AKT expression level among cancer cells in our study.

Although no study has examined sulforaphane levels in the ovaries or ovarian tumors, sulforaphane may be readily absorbed. A single dose of 200 μM broccoli sprout isothiocyanates (77.2% sulforaphane) was rapidly absorbed and reached a maximum concentration of approximately 2 μM in the plasma and erythrocytes of healthy human volunteers [37]. Sulforaphane was also rapidly bioavailable in rats, in which oral gavage of 50 μM sulforaphane performed twice reached a maximum plasma concentration of approximately 20 μM [38]. Thus, previous studies indicate that sulforaphane is readily absorbed and can reach a dose observed to be effective in our study.

MAPKs play important roles in mediating cellular signaling. ERK signals have been demonstrated to be important intracellular mediators of the cell-cycle arrest induced by 25 μM sulforaphane in Caco-2 cells [24]. We also observed an increase in phosphorylation of ERK and p38 MAPK after treatment with sulforaphane (Figure 2(A)). However, activation of ERK and p38 MAPK by sulforaphane was not dose dependent (Figure 2(A)). SB203580 and PD98059, specific inhibitors of p38 and ERK, respectively, failed to abrogate the effect of sulforaphane (Figure 2(B,C)). Thus, the inhibition of cell growth induced by sulforaphane treatment did not appear to be mediated through MAPK pathways in our study. Yeh and Yen also reported that sulforaphane-induced activation of the ERK, p38, and JNK MAPK pathways occurred at a non-toxic dose (20 μM) in HepG2 cells [27]. They showed that these activated MAPK pathways were responsible for the induction of nuclear factor (erythroid-derived 2)-like 2 (Nrf2)-mediated metallothionein protein [27]. In addition, p38 or ERK MAPK was involved in the induction of antioxidant response element-mediated heme oxygenase-1 [25] and Nrf2-dependent enzymes [39] during sulforaphane treatment. Therefore, in some cell lines, MAPK pathways induced by sulforaphane may be related to the induction of phase II and antioxidant enzymes at non-cytotoxic doses.

Sulforaphane has been reported to induce cell death in some cancer cells through the generation of ROS, based on the finding that the ROS scavenger NAC prevented sulforaphane-induced inhibition of cell growth [20–22]. We also demonstrated that the effect of sulforaphane on cell growth is abrogated by pretreatment with NAC (Figure 3(A)). However, sulforaphane did not appear to markedly increase the generation of intracellular ROS in our study (Figure 4(A)). Previous studies reported an increase in intracellular ROS after treatment with a cytotoxic dose (greater than 20 μM) of sulforaphane [21,22]. In those studies, intracellular ROS levels were estimated by changes in the fraction of cells that contained a high level of DCF using flow cytometry. In contrast, we used a plate reader to analyze fluorescence intensity changes. Flow-cytometry measurements may be more sensitive in determining intracellular ROS levels, especially when a certain fraction of cells is more susceptible to sulforaphane-induced ROS generation. However, one previous study used higher doses (10 mM) of NAC [21] compared to the 2 mM used in our study, suggesting that NAC may play different roles in the sulforaphane-mediated effects observed in different studies.

In addition, pretreatment with the ROS-scavenging antioxidants BHA and Trolox had no effect on the sulforaphane-mediated inhibition of cell growth (Figure 4(C,D)). In contrast, another thiol-reducing agent, DTT, had a similar effect to NAC treatment (Figure 4(B)). Moreover, the thiol-oxidizing agent diamide enhanced sulforaphane-induced inhibition of cell growth (Figure 5(A)) and sulforaphane-induced cell death (Figure 6). Thus, thiol reduction by NAC rather than inhibition of ROS may be important for the decrease in sulforaphane-induced inhibition of cancer cell growth observed in our study. Treatment with NAC and diamide was less effective in fibroblasts than in cancer cells in the prevention or enhancement of sulforaphane-induced growth inhibition (Figures 3 and 5), probably because single treatment of sulforaphane was less effective in the induction of growth inhibition in fibroblasts.

It has been suggested that caution should be applied when NAC is used as an antioxidant since it also possesses reducing capability via its thiol-disulfide exchange activity independent of its antioxidative or free radical scavenging properties [40,41]. NAC has been shown to prevent apoptosis induced by methyl-2-cyano-3,12-dioxooleana-1,9-dien-28-oate [42]. However, its ability to induce apoptosis is mediated through a direct interaction with thiol-containing components and does not involve ROS generation in human lung cancer cells [42]. Sulforaphane also possesses sulfhydryl-modifying activity through reaction of its isothiocyanate moiety with thiol groups in proteins [43]. In our study, sulforaphane-mediated growth inhibition was greatly attenuated by thiol-reducing agents (e.g. NAC and DTT) and was enhanced in the presence of thiol-oxidizing agents (e.g. diamide). This observation suggests that the decrease in cell viability or proliferation induced by sulforaphane in our study may be due to a direct reaction between sulforaphane and cysteine residues of protein(s) or alterations in the cellular thiol redox state that lead to alterations in cell proliferation or death.

There has been concern regarding the use of antioxidants such as NAC in conjunction with cancer therapeutic agents [44]. The use of NAC has been reported to increase tumor growth in animal models [45] and to alter the efficacy of cancer therapy [46]. NAC was shown to be independently associated with worse recurrence-free and overall survival in breast cancer patients who received NAC as an adjuvant chemotherapy [47]. In parallel with these findings, our study indicated that supplementation with NAC can abrogate the therapeutic effect of sulforaphane, and that this may be mediated by its thiol-reducing capability.

## Conclusions

Sulforaphane effectively reduced cell viability and/or growth in ovarian cancer cells with different genetic backgrounds in our study. Sulforaphane may act at least partly by interacting with protein thiols and/or altering the cellular thiol redox status, thus changing the expression levels of proteins important for cell survival and proliferation. The therapeutic effect of sulforaphane can be abrogated by thiol-reducing agents such as NAC supplements but not by the ROS-scavenging antioxidants abundant in vegetables. Further study is required to determine the underlying mechanisms involved in thiol modification (binding to target proteins) or thiol redox state changes induced by sulforaphane and how these changes may lead to alterations in the expression of cell proliferation- and/or cell survival-related proteins.

## Supplementary Material

Rev_FNR_supporting_information.docx
