# Supplementary material for: Thiol-reducing agents prevent sulforaphane-induced growth inhibition in ovarian cancer cells
Source: Food Nutr Res. 2017 Aug 28;61(1):1368321. doi: 10.1080/16546628.2017.1368321 (PMC5614215; doi:10.1080/16546628.2017.1368321)
Supplement: Rev_FNR_supporting_information.docx [file ZFNR_A_1368321_SM7934.docx]

**Supporting information**

**
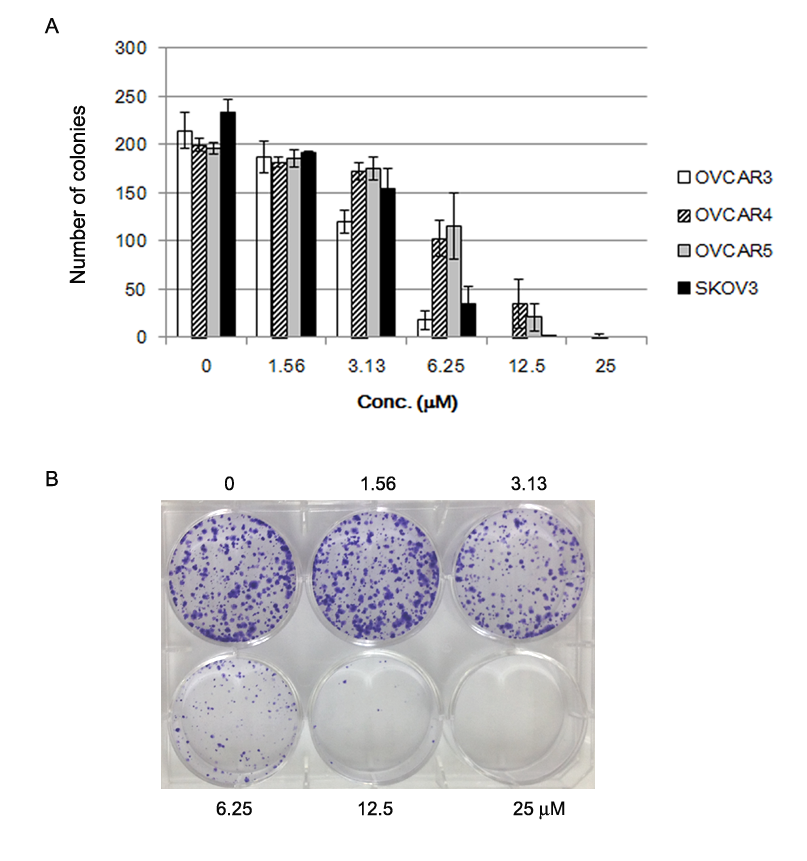
**

**Figure S1.** Inhibition of cell viability by treatment with sulforaphane in ovarian cancer cells using a colony formation assay. OVCAR3, OVCAR4, OVCAR5, and SKOV3 cells were cultured in 6-well plates overnight and treated with 0 to 25 μM sulforaphane for 14 days. Cell viability was assessed using a colony formation assay. (A) The total number of colonies formed was counted. Values are mean ± SE, n=3. (B) Images shown are representative of colonies of OVCAR3 cells formed in the presence of different concentrations of sulforaphane.


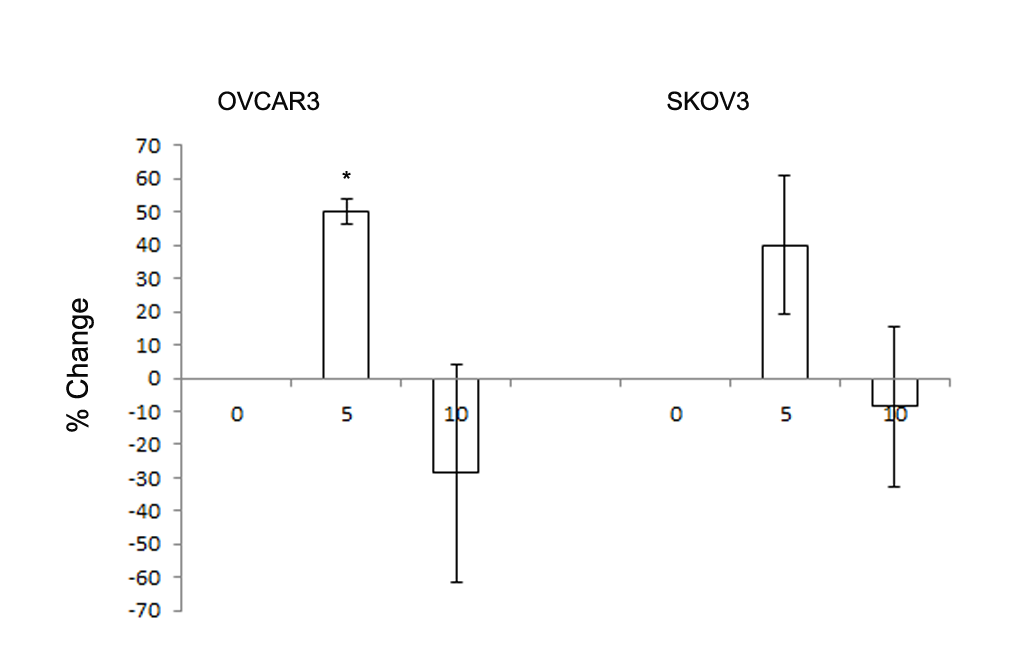


**Figure S2.** Effect of sulforaphane on thioredoxin reductase activity. Thioredoxin reductase activity was measured 24 hr after treatment of OVCAR3 and SKOV3 cells with 5 and 10 μM sulforaphane. Values are mean ± SE, n=3. Asterisk (*) represents significant difference (p < 0.05) in activity compared to untreated control.
